# Rare disease patient matchmaking: development and outcomes of an internet case-finding strategy in the Undiagnosed Diseases Network

**DOI:** 10.1186/s13023-021-01825-1

**Published:** 2021-05-10

**Authors:** Kimberly LeBlanc, Emily Glanton, Anna Nagy, Jorick Bater, Tala Berro, Molly A. McGuinness, Courtney Studwell, Undiagnosed Diseases Network, Matthew Might

**Affiliations:** 1 Department of Biomedical Informatics, Harvard Medical School, Boston, MA USA; 2 Department of Nutrition, Harvard T.H. Chan School of Public Health, Boston, MA USA; 3 Department of Medicine, Brigham and Women’s Hospital, Boston, MA USA; 4 Bass Center for Childhood Cancer and Blood Diseases, Stanford Children’s Health, Palo Alto, CA USA; 5 Department of Pathology, Brigham and Women’s Hospital, Boston, MA USA; 6 Hugh Kaul Precision Medicine Institute, University of Alabama at Birmingham, Birmingham, AL USA

**Keywords:** Rare disease, Gene discovery, Matchmaking, Data sharing, Diagnostic odyssey

## Abstract

**Background:**

Although clinician, researcher, and patient resources for matchmaking exist, finding similar patients remains an obstacle for rare disease diagnosis. The goals of this study were to develop and test the effectiveness of an Internet case-finding strategy and identify factors associated with increased matching within a rare disease population.

**Methods:**

Public web pages were created for consented participants. Matches made, time to each inquiry and match, and outcomes were recorded and analyzed using descriptive statistics. A Poisson regression model was run to identify characteristics associated with matches.

**Results:**

385 participants were referred to the project and 158 had pages posted. 579 inquiries were received; 89.0% were from the general public and 24.7% resulted in a match. 81.6% of pages received at least one inquiry and 15.0% had at least one patient match. Primary symptom category of neurology, diagnosis, gene page, and photo were associated with increased matches (*p* ≤ 0.05).

**Conclusions:**

This Internet case-finding strategy was of interest to patients, families, and clinicians, and similar patients were identified using this approach. Extending matchmaking efforts to the general public resulted in matches and suggests including this population in matchmaking activities can improve identification of similar patients.

**Supplementary information:**

The online version contains supplementary material available at 10.1186/s13023-021-01825-1.

## Background

Genomic testing has revolutionized Mendelian disease diagnosis [1–4]. Exome and genome sequencing are able to uncover the causes of rare and previously undiagnosed conditions, ending the diagnostic process for patients and their families. However, genes and variants of uncertain significance are frequently identified through genomic testing, leaving families without answers and many rare conditions undiagnosed.

In order to confidently associate genes and variants with disease, often individuals with the same or similar phenotype and variant must be identified [5]. This genomic matchmaking process involves identifying cases with similar phenotypic features and genomic findings. The Matchmaker Exchange has emerged as a resource for clinicians and researchers to facilitate case matching [6]. This resource connects databases of genotypic and phenotypic data using a common application programming interface (API). The Matchmaker Exchange effort has raised awareness of the importance of data sharing and use of this platform has improved case matching and discovery of new Mendelian conditions [7]. Despite these advancements, genomic matchmaking remains a limiting factor for rare disease diagnosis, partly due to the limited number of providers utilizing matchmaking platforms [5] and protection of scientific knowledge.

Involving patients and families in the genomic matchmaking process has the potential to increase the size of the matchmaking network and improve the likelihood that a similar case will be discovered [5]. Often, patients and families are experts in their conditions and can provide unique knowledge beneficial in this search [8, 9]. Patient- and family-facing platforms like MyGene2 and GenomeConnect have been created to promote patient-led matching and provide increased control over data sharing [1, 10, 11]. However, currently data entered by patients and families on these platforms is not shared through Matchmaker Exchange, limiting matchmaking capabilities between patients, families, clinicians, and researchers [7].

In order to share data more broadly and find other similar individuals, many patients and families have turned to blogs, social media platforms, and automated strategies like Google alerts to identify similar individuals [5, 9, 12]. Several of these patient- and family-led efforts have been successful in identifying other individuals with the same genetic condition and have led to novel rare disease diagnoses [9, 12]. In a study focused on parental perspectives on Internet and social media use, the majority of participants noted that they felt comfortable sharing details about their child’s condition and genetic results on these types of platforms [13]. Turning to the Internet and social media for research and support appears to be a common practice among those impacted by rare conditions [14].

Although there seems to be interest in the undiagnosed and rare disease community to use the Internet and social media as resources for learning and connection, barriers to this type of engagement exist. For instance, language barriers or lack of Internet access may prevent review and vetting of information, or individuals may lack expertise or connection to reach key clinicians and researchers [1, 5, 15]. These barriers may prevent some from engaging in these practices even if there is interest, limiting matchmaking possibilities.

Improvements in matchmaking have the potential to end the diagnostic process for many and connect similar individuals with one another for support. Although clinician, researcher, and patient resources for matchmaking exist, finding other similar patients is still a limiting factor for rare disease diagnosis. A broad matchmaking strategy involving patients, families, clinicians, researchers, and those connected to them could enable all to work jointly to identify individuals with the same gene of interest or condition. By leveraging the fact that members of each of these groups turn to the Internet for information, we aimed to pilot an Internet-based case-finding strategy in the Undiagnosed Diseases Network (UDN), a research study funded by the National Institutes of Health (NIH) to provide diagnoses to individuals with undiagnosed conditions [16, 17]. The goals of our study were to: (1) develop an Internet case-finding strategy in collaboration with UDN patients, families, and clinicians, (2) ascertain the effectiveness of finding other similar individuals using this method, and (3) identify factors associated with matches.

## Methods

### Participants

UDN participants and parents/guardians of participants were invited to take part in this study. To be eligible, participants were required to have undergone evaluation through one of the UDN clinical sites and determined by the site to potentially benefit from the sharing of information publicly. The site’s decision was based on genotypic and phenotypic findings that could benefit from matching with similar individuals. All participants and parents/guardians who expressed interest and met the inclusion criteria were contacted regarding participation from April 14, 2016 to April 13, 2020 by research assistants at the UDN Coordinating Center, which manages network-wide activities. The study protocol was approved by the NIH Institutional Review Board (IRB) (protocol 15-HG-0130).

### Procedures

Invitation emails were sent by Coordinating Center research assistants to the participants’ primary email addresses recorded in the UDN database. Participants without email addresses were contacted using the primary phone number. Individuals who did not respond were contacted up to two additional times. Participants and parents/guardians were consented over the phone and written consent forms were obtained for individuals enrolled between April 14, 2016 and July 11, 2018. In July 2018, the NIH IRB approved a waiver of written informed consent for the study. Between July 12, 2018 and April 13, 2020, participants and parents/guardians were provided with a consent form summarizing the study and confirmed interest by email or phone. A genetic counselor and research assistant were available to answer questions regarding participation. A genetic counselor on the project team fluent in Spanish was able to discuss participation with Spanish-speaking individuals. A phone translation service was used for communication with individuals who did not speak English or Spanish. Participation in the study was voluntary.

Web pages for consented participants were created by Coordinating Center research assistants by gathering information from the UDN database and were reviewed by genetic counselors at the Coordinating Center, clinical site providers, and the participant or parent/guardian. The web pages included the following information as applicable: genetic variants; signs and symptoms using plain language with the corresponding human phenotype ontology (HPO) term in parentheses; past medical history; past and current treatments, procedures, and medications; age; sex; pictures and/or videos, if participant consented; and contact information for the Coordinating Center. Pages included both a narrative description as well as listed information. For each gene of interest listed on the page, a separate gene page was created and linked to the participant webpage. This gene page included variant information and links to external sites with information about the gene. Google alerts for all gene names of interest were created; search engine optimization techniques, such as linking to external sites and adding gene names to the page name, were used to ensure that the pages were found. Once the pages were posted on the UDN public website [18], links to the pages were shared on UDN social media accounts if the participant and/or parent/guardian consented to such sharing. Updates to pages could be requested by the participant, parent/guardian, and clinical site as needed. Examples of the layout of the web pages can be found on the UDN website [18].

Contacts regarding the web pages were triaged and recorded by genetic counselors at the Coordinating Center. For potential matches, efforts were made to collect additional information. Conversations between inquirers, clinical sites, and participants or parents/guardians were facilitated by the Coordinating Center. Participants and parents/guardians were asked if they would like to communicate with inquirers before they were connected. The amount of time spent processing each inquiry, how many matches were made, the length of time to each inquiry and match, and outcomes were recorded.

Matches were initially coded by two genetic counselors on the project team. Matches were coded as inquiries relating to the gene of interest (“gene-only match”), diagnosis of interest (“diagnosis-only match”), or another individual with a variant in the gene of interest and similar symptoms (“patient match”). A third genetic counselor on the project team independently coded all matches. Codes were reviewed and discussed, and disagreements were resolved amongst the three team members.

### Data analysis

Data collected from April 14, 2016 to April 13, 2020 were analyzed. Quantitative data were analyzed using descriptive statistics, including calculation of means, medians, and percentages. Data distribution was visualized using JMP Pro 14.1 [19]; means were selected for normally distributed data and medians were selected for non-normally distributed data. The Flesch-Kincaid Grade Level test was used to assess readability of narrative page descriptions after removing parenthetical HPO terms, which were included as technical terminology after a plain-language description [20].

A Poisson regression model [21] was run to identify characteristics associated with matches. Characteristics considered as potential predictor variables included: (1) participant demographics (pediatric or adult status, language, sex, race, ethnicity, and primary symptom type of neurology), ((2) description length and readability (word count and Flesch-Kincaid Grade Level), (3) the presence or absence of information on a page (current treatment, prior treatment, considered treatment, previously considered treatment, diagnosis, photo, and total sections filled), (4) number of genetic variants (gene page count, presence or absence of a linked gene page), and (5) page sharing (whether the page was shared on social media). For the purpose of this analysis, pages that had genes added after initial posting were considered as pages with genes. Time between the page being posted and the end of study was used as an offset term to account for varying exposure times between web pages. Given the large number of potential predictors, Lasso [21], a penalized regression, was used to choose variables to prevent overfitting. The Lasso-chosen variables were run with the Poisson regression model. A *p*-value less than or equal to 0.05 in the Poisson model was considered the threshold for final selection. All analyses were performed on R Software version 4.0.2 for Mac OS X.

## Results

### Demographic characteristics

Demographic characteristics are presented in Table 1. A total of 385 participants were referred to the project by their clinical site after expressing interest. Participants had a median (IQR) age of 10 (4, 24) and were predominantly female (51.2%, 197/385), white (84.2%, 324/385), and non-Hispanic/Latinx (75.8%, 292/385). Of all the participants referred to the project, 96.6% (372/385) were English-speaking, 3.1% (12/385) were Spanish-speaking, and 0.3% (1/385) were Kurdish-speaking. A total of 158 participants (median (IQR) age 9 (4, 17.25); 48.7% (77/158) female, 81.6% (129/158) white, 76.6% non-Hispanic/Latinx (121/158)) had pages posted. Of the participants with pages posted, 97.5% (154/158) were English-speaking and 2.5% (4/158) were Spanish-speaking.

**Table 1:** Demographic characteristics

Demographics	Total (N = 385)	Pages not posted (N = 227)	Pages posted (N = 158)
Age			
Age in years (median (IQR))	10 (4, 24)	11 (4, 31)	9 (4, 17.25)
Pediatric	66.7% (257)	60.8% (138)	75.3% (119)
Adult	33.3% (128)	39.2% (89)	24.7% (39)
Sex, %, (n)			
Female	51.2% (197)	52.9% (120)	48.7% (77)
Male	48.6% (187)	47.1% (107)	50.6% (80)
Other	0.2% (1)	0% (0)	0.6% (1)
Race, %, (n)			
White	84.2% (324)	85.9% (195)	81.6% (129)
More than 1 race	6.0% (23)	3.5% (8)	9.5% (15)
Asian	4.4% (17)	5.3% (12)	3.2% (5)
Black or African American	2.3% (9)	1.8% (4)	3.2% (5)
Other race (not specified)	1.8% (7)	2.2% (5)	1.3% (2)
American Indian or Alaska Native	0.5% (2)	0.9% (2)	0% (0)
Race not provided	0.8% (3)	0.4% (1)	1.3% (2)
Ethnicity, %, (n)			
Not hispanic/Latinx	75.8% (292)	75.3% (171)	76.6% (121)
Hispanic/Latinx	13.2% (51)	12.8% (29)	13.9% (22)
Ethnicity not provided or unknown	10.9% (42)	11.9% (27)	9.5% (15)
Language, %, (n)			
English	96.6% (372)	96.0% (218)	97.5% (154)
Spanish	3.1% (12)	3.5% (8)	2.5% (4)
Kurdish	0.3% (1)	0.4% (1)	0% (0)

### Web page development

Of the participants and parents/guardians of participants who expressed interest in the project, 97.4% (375/385) were contacted by the Coordinating Center to provide additional information about participation. Ten individuals were not contacted because contact information was not available or benefits of participation needed to be clarified with the clinical site. Of those who were contacted, 36.3% (136/375) did not respond to emails or phone calls made by the Coordinating Center. These individuals may have (1) opted to not participate, or (2) decided that they did not have the time to work with the Coordinating Center to review draft pages. They remain in the UDN system and can opt to participate at a later date.

Of the participants and parents/guardians who expressed continued interest, pages were drafted for 79.0% (189/239). The primary reasons pages were not drafted were: (1) families were lost to follow-up during the consenting process, and (2) a diagnosis was made in the interim period. Pages were posted for 158 participants. Reasons pages were not posted at the time of the analysis included: approval pending from the participant or parent/guardian (71.0%, 22/31), approval pending from the clinical site (19.4%, 6/31), diagnosis made that decreased potential benefit (6.5%, 2/31), and pending publication (3.2%, 1/31).

For pages posted, 96.2% (152/158) were shared on social media and 87.3% (138/158) included photographs. Diagnoses were not present for 61.3% (97/158) of the pages and 55.7% (88/158) included genes of interest. Narrative descriptions included a mean word count of 145 words (SD ± 52.4) and had a mean Flesch-Kincaid Grade Level of 11.4 (SD ± 2.3).

### Inquiries and outcomes

From April 14, 2016 to April 13, 2020, 579 inquiries were received and triaged by genetic counselors at the Coordinating Center. The median number of inquiries per page (IQR) was 2 (1, 5) and 81.6% (129/158) of pages received more than one inquiry. The median (IQR) number of days between page posting and first inquiry was 3 days (0, 51.75). The mean length of time spent processing each inquiry was less than 15 min.

**Fig. 1:**
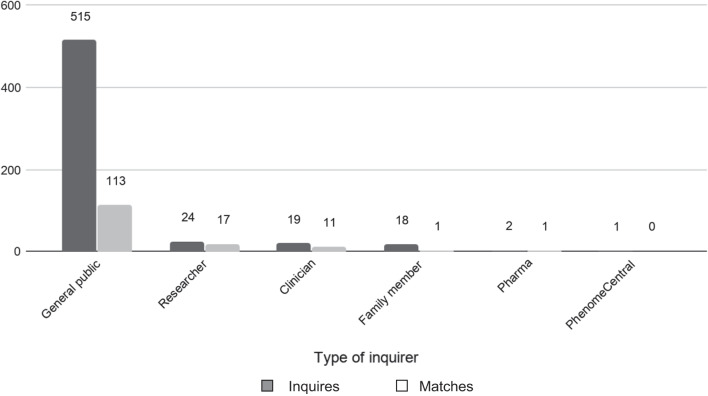
Type of inquirer and matches

**Fig. 2:**
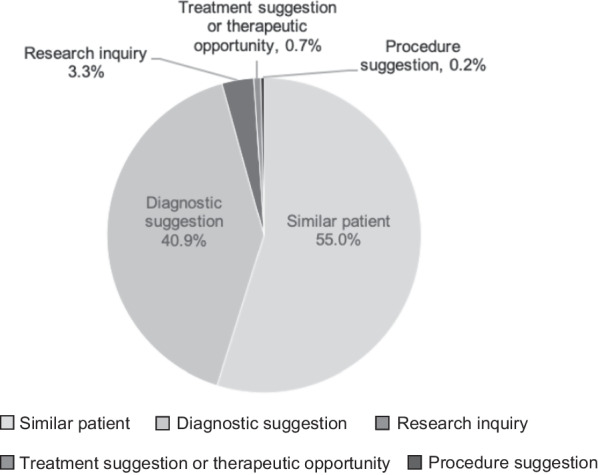
Type of inquiry

Of all the inquiries, 89.0% (515/579) were from the general public (included patients and family members impacted by rare and undiagnosed conditions), 4.2% (24/579) were from researchers, 3.3% (19/579) were from clinicians, 3.1% (18/579) were from individuals with family members in the UDN, 0.4% (2/579) were from pharmaceutical company employees, and 0.2% (1/579) were from representatives from PhenomeCentral (Fig. 1). Of note, two of the clinician inquiries were from members of the UDN. Types of inquiries received are presented in Fig. 2. The majority of inquiries were regarding a similar patient (55.0%, 318/579) or diagnostic suggestion (40.9%, 237/579). The remainder of contacts were regarding a research inquiry (3.3%, 19/579), therapeutic suggestion or opportunity (0.7%, 4/579), or procedure suggestion (0.2%, 1/579).

Several of the 579 inquiries resulted in multiple outcomes; for example, some inquiries were connected with both the clinical site and other families. The most common inquiry outcomes included sending the diagnostic suggestion to the clinical site for review (n = 222) and connecting the inquirer with the clinical site to discuss the similar patient or diagnostic suggestion (n = 65). One hundred and six inquiries resulted in the inquirer being connected with families (n = 58) or resources (n = 48). In several cases, families communicated directly through comments on the UDN Facebook page or through sharing on their personal social media accounts. Ninety-nine inquiries were determined to not be a match by the Coordinating Center or clinical site. Additional information was requested by the Coordinating Center in 33 cases and, for individuals who met UDN inclusion criteria, information about applying to the UDN was sent (n = 66). For 93 inquiries, no action was taken beyond Coordinating Center review.

Overall, 24.7% (143/579) of inquiries resulted in a match. All but 9 of these inquiries resulted in an action by the Coordinating Center. Of the matches, 58.0% (83/143) were classified as a gene-only match, 37.8% (54/143) as a patient match, and 4.2% (6/143) as a diagnosis-only match (Additional file 1). The matches per inquiries rate was 70.8% (17/24) for researchers, 57.9% (11/19) for clinicians, 50% (1/2) for pharmaceutical company employees, 21.9% (113/515) for the general public, 5.6% (1/18) for family members of UDN participants, and 0% (0/1) for PhenomeCentral representatives (Fig. 1). The median (IQR) number of days between page posting and first match was 73 days (12.75, 217.25) (range: 0–1304 days). Of the 158 pages, 29.7% (47/158) had at least one match of any type and 15.0% (23/158) had at least one patient match.

### Characteristics associated with matches

The variables chosen by the Lasso-Poisson model were: primary symptom type of neurology, description Flesch-Kincaid Grade Level, and presence of a confirmed diagnosis, gene page, and photo. The Poisson model selected the following variables to be the most associated with matches (*p* ≤ 0.05): primary symptom type of neurology and presence of a diagnosis, gene page, and photo.

### Illustrative cases

As part of this study, eight patients with Neurodevelopmental disorder with epilepsy, cataracts, feeding difficulties, and delayed brain myelination (NECFM) (OMIM #617393) and five patients with Neurodevelopmental disorder with regression, abnormal movements, loss of speech, and seizures (NEDAMSS) (OMIM # 618088) were identified (Additional file 1). All but one of the inquiries regarding patients with NECFM and NEDAMSS were from family members. Families were subsequently connected with UDN research teams and one another to discuss ongoing research and support options. In both cases, families opted to form online support groups and are actively engaged in and funding research. Patients identified through the web pages were also included in UDN publications describing these novel conditions [22, 23].

## Discussion

Identifying patients with the same condition remains an obstacle for rare disease diagnosis. Making matchmaking efforts accessible to the general public has the potential to increase the size of the matchmaking network and likelihood of finding similar patients. In this study, we found that there was interest amongst patients and families impacted by rare and undiagnosed conditions to participate in a public Internet case-finding strategy. This method was successful in identifying other individuals with the same genetic condition. Mention of a diagnosis, link to a gene page, and presence of a photo increased total matches in this sample. These results suggest that broad Internet case-finding strategies have the potential to identify similar patients and offer a framework for replication in other settings.

In partnership with UDN patients, families, and clinical sites, pages were created on the UDN website. After expressing initial interest to clinical site coordinators, the majority of participants and parents/guardians expressed continued interest to the Coordinating Center. In particular, parents of pediatric UDN participants seemed especially interested in this data sharing approach. Consistent with prior studies [13, 14], individuals were comfortable sharing information on the Internet and social media, including photographs. Although the vast majority of participants were English-speaking, individuals who did not speak English were able to participate, which may not be possible with other matchmaking platforms. Participation also did not require having Internet access or creating an account, which may be barriers for some [1, 5, 15].

Based on the number of inquiries received from members of the general public, there seems to be broad interest in this case-finding approach. Interestingly, although all UDN clinicians and researchers have access to an internal UDN database with case information and to the Matchmaker Exchange [6], two UDN clinicians inquired about pages through the UDN website. This supports the notion that having pages appear in Internet search results is accessible for both the general public and clinicians. We also found that external rare disease groups had interest in sharing UDN pages on their social media accounts. For example, the Genetic and Rare Diseases Information Center [24] shared page descriptions in Spanish on their Facebook page and Comitato I Malati Invisibili, a non-profit focused on rare and undiagnosed conditions in Italy, shared page descriptions in Italian on their Twitter account and website.

Not only does there appear to be broad interest in this case-finding strategy, our results indicate that it is effective in identifying similar patients. Notable examples include connections with other patients with NECFM and NEDAMSS. These connections are not only crucial in associating genes and variants with disease, they can also lead to the establishment of patient communities. In the NECFM and NEDAMSS cases, families were able to form online groups to exchange information and support. This type of engagement with others who have family members with a similar condition is a known need of families impacted by rare genetic conditions [13, 25]. Since connecting, these families have also played an active role participating in and funding ongoing research related to these novel conditions.

Overall, we found that pages with a mention of a diagnosis, link to a gene page, and presence of a photo increased total matches in this sample. This suggests that future attempts at patient matchmaking using this type of approach may benefit from the inclusion of these variables on pages. For example, inclusion of a photo and a gene of interest on patient pages may increase the match rate for other similar initiatives. Also, the match rate was significantly higher for inquiries received from clinicians and researchers than the general public. This suggests that limiting matchmaking efforts to clinician and researcher cohorts could decrease the burden of reviewing inquiries that do not result in matches. However, excluding the general public from these activities prevents any matches from this sizable group. In total, 21.9% of matches came from the general public in this study and the average inquiry processing time was less 15 min, suggesting there is still a benefit to including this population in matchmaking efforts.

Although the pages were created primarily to find other similar patients, a large proportion of inquiries received were diagnostic suggestions. These suggestions ranged from single gene disorders to more common conditions with complex etiologies. To our knowledge, diagnostic suggestions submitted did not result in new diagnoses for UDN participants; however, it demonstrates that there is interest in the general public to be involved in the diagnostic process. This interest may be motivated by knowledge of the extended diagnostic odyssey for many rare disease patients and misdiagnoses during this process [26]. The mainstream media has recognized, and capitalized on, the attraction to diagnostic crowdsourcing. Article series and television programs have been developed that focus on this concept [27]. Technical platforms have also been built to support diagnostic contributions from a variety of individuals [28].

In addition to the unexpected outcome of diagnostic suggestions, many inquiries received were from individuals with undiagnosed conditions who ended up applying to the UDN. These individuals were initially making contact about the pages, but many had interest in being part of the network as well. While we did not intend to use the pages as a recruitment strategy, the project did result in the submission of numerous applications that were eventually accepted.

This study had several limitations. First, participants were primarily white, non-Hispanic/Latinx, and English-speaking, which impacts the generalizability of the results. Reasons for declining to participate were not collected, so we are unable to draw conclusions about obstacles to participation. Although a high likelihood of matching was not used as an inclusion criterion, given the voluntary nature of the study, it is possible participants who enrolled had a higher likelihood of matching that those in the broader UDN cohort. With that said, the number of matches, particularly patient and diagnosis matches, was likely underestimated since the interaction with inquirers was limited and follow-up information was not obtained. In future studies, more information could be collected from inquirers at the time of initial contact and updates could be gathered over time. Although the participants in this study were comfortable with broad data sharing, we did not survey all participants and families in the UDN regarding their feelings surrounding public data sharing. Since not all individuals would be supportive of broad data sharing, this strategy is limited to those comfortable with public sharing. In addition, these pages were only available on the UDN website in English so those without Internet access or whose primary language was not English may not have been able to access them. In the match characteristic analysis, the small sample size may have inflated standard errors or reduced power of certain predictor variables that did not have enough samples between categories. Using a Poisson model also has the assumption that mean equals variance, which could be too strong in cases of overdispersion.

## Conclusions

Overall, we found that there was interest amongst patients and families impacted by rare and undiagnosed conditions and their clinicians to partner in the development of a public Internet case-finding strategy. Our findings support the need for additional work in expanding patient matchmaking to include patients, families, clinicians, researchers, and the general public. This study was done in a small cohort of individuals through the UDN; however, a larger scale effort using a similar strategy may be warranted. In the future, public web pages which are accessible to individuals with varying levels of expertise may be useful tools to increase matchmaking. Future research efforts could also focus on how best to incorporate factors found to increase match rates in this study. In summary, matchmaking is a crucial part of the diagnostic process for many individuals with rare conditions; broadening these efforts to the general public through Internet case-finding strategies has the potential to improve the process and connect more patients and families with one another.

### Supplementary information

**Additional file 1**: Table of inquiries and matches per web page.

## Data Availability

All web pages are available on the UDN public website. Information regarding the number and type of inquiry per page is available in the Additional file 1. The additional datasets used and/or analyzed during the current study are available from the corresponding author on reasonable request.

## Abbreviations

API: application programming interface
HPO: human phenotype ontology
IRB: Institutional Review Board
NIH: National Institutes of Health
NECFM: neurodevelopmental disorder with epilepsy, cataracts, feeding difficulties, and delayed brain myelination
NEDAMSS: neurodevelopmental disorder with regression, abnormal movements, loss of speech, and seizures
UDN: Undiagnosed Diseases Network
